# Morphological and Molecular Characterization of *Kochinema farodai* Baqri and Bohra, 2001 (Dorylaimida: Nordiidae) from California, with the First Molecular Study and an Updated Taxonomy of the Genus

**DOI:** 10.3390/ani10122300

**Published:** 2020-12-04

**Authors:** Sergio Álvarez-Ortega

**Affiliations:** Departamento de Biología y Geología, Física y Química Inorgánica, Universidad Rey Juan Carlos, Campus de Móstoles, 28933 Madrid, Spain; sergio.aortega@urjc.es

**Keywords:** 28S rRNA, compendium, diagnosis, key, SEM, *species incertae sedis*, systematics

## Abstract

**Simple Summary:**

In this paper, a population of a free-living nematode collected from Northern California (USA) is identified and studied. The aim of the present work is to characterize a Californian population of the nematode genus *Kochinema*, with morphological and molecular methods. In addition, a revision of this genus is also provided including an updated diagnosis, a list of species, a key to species identification, and a compendium of their main morphometrics and distribution data.

**Abstract:**

This paper deals with the morphological and molecular characterization of *Kochinema farodai* Baqri and Bohra, 2001, with an integrative approach. The finding of *K. faroidai* in California is a remarkable biogeographical novelty, as it is the first American record of the species. Molecular data herein obtained represent the first molecular study of the genus *Kochinema*. Furthermore, scanning electron microscopy (SEM) observations of a member of *Kochinema* are provided for the first time. Additionally, this contribution provides new insights into the phylogeny and taxonomy of the nematode genus *Kochinema*. A brief historical outline of the matter is presented. Then, the morphological pattern of the genus is revised and illustrated, the anterior position of amphids, whose opening is located on lateral lip, being its most relevant diagnostic feature. The phylogenetic analysis inferred from D2–D3 expansion segments of 28S rRNA gene shows that *Kochinema* clustered together with other dorylaimid species characterized by the absence of *pars refringens vaginae* and that it does not share a recent common ancestor with other members of the family Nordiidae. A likely polyphyly of the family Nordiidae is confirmed. Finally, an updated taxonomy of the genus is proposed, including a revised diagnosis, a list of species, a key to species identification, and a compendium of their main morphometrics and distribution data.

## 1. Introduction

The genus *Kochinema* Heyns, 1963, is a very interesting and rare (infrequent) dorylaimid taxon. *Kochinema* has a narrow distribution, with occurrence only in Africa and Asia [1]. Among other features, its morphological pattern is characterized by the presence of a lip region wider than adjacent body part, anterior position of amphids, odontostyle slender and longer than lip region diameter, small odontostyle aperture, absence of *pars refringens vaginae*, and conical tail with rounded terminus. Currently, eight valid species are listed under *Kochinema* [1], but most of these species were described based on only a few specimens (see Table S1). This nematode genus has never been the subject of a revision of its taxonomy, and to date, there are no molecular data available of the genus which allow the elucidation of the relationships of *Kochinema* with its closest taxa.

During nematological surveys conducted during the spring–summer of 2011 in natural areas of Northern California (USA), several specimens belonging to *Kochinema* were collected. The morphological and molecular characterization of this new population of *Kochinema* from California provided new data that were used to elucidate the taxonomy and phylogeny of this rare nematode genus.

## 2. Materials and Methods

### 2.1. Sampling, Extraction, and Morphological Identification

Nematode population used in this study was collected in a natural area from El Dorado County, California. Nematodes were extracted from soil samples by sieving and sucrose-centrifugation technique (specific gravity = 1.18), somewhat modified, according to Barker [2]. The specimens were relaxed and killed by heat, fixed in 4% formaldehyde, and processed to anhydrous glycerin following Siddiqi’s [3] method. Finally, the nematodes were mounted on permanent glass slides to allow handling and observation under light microscopy (LM).

Measurements of specimens were taken using a light Olympus BX51 microscope (Olympus, Tokyo, Japan) equipped with differential interference contrast. Morphometrics included de Man’s indices and standard measurements. Some of the best-preserved specimens were photographed with a Canon EOS 250D digital camera (Canon, Tokyo, Japan). Digital images were edited using Adobe^®^ Photoshop^®^ CS.

After their examination and identification, a few specimens preserved in glycerin were selected for observation under scanning electron microscopy (SEM) following the protocol by Álvarez-Ortega and Peña-Santiago [4]. The nematodes were hydrated in distilled water, dehydrated in a graded ethanol and acetone series, critical point-dried, coated with gold, and observed with a Zeiss Merlin microscope.

Morphological and morphometrical analysis on all known species of *Kochinema* is based on a comprehensive compilation and revision of available literature on this matter.

### 2.2. DNA Extraction, PCR, and Sequencing

DNA was extracted from a single individual using the proteinase K protocol. Nematode material was transferred to an Eppendorf tube containing 30 μL double distilled water, 3 μL PCR buffer (Qiagen, Hilden, Germany), and 2 μL proteinase K (600 μg mL^−1^) (Qiagen). The tubes were incubated at 65 °C (1 h) and then at 95 °C (15 min). PCR and sequence protocols were as described by Álvarez-Ortega et al. [5]. The primers used for amplification of the D2–D3 region of 28S rDNA gene were the D2A (5′-ACAAGTACCGTGAGGGAAAGTTG-3′) and the D3B (5′-TCGGAAGGAACCAGCTACTA-3′) primers [6].

PCR products were purified using the QIAquick PCR purification Kit (Qiagen) and used for direct sequencing. The sequencing reactions were performed at ”Davis Sequencing”, Davis, CA, USA. The newly obtained sequence was submitted to the GenBank database under accession number: MW243335.

### 2.3. Phylogenetic Analyses

The newly obtained sequence was aligned with other sixty-five D2–D3 expansion segments of 28S rRNA gene sequences available in GenBank using ClustalX 1.83 [7]. Outgroup taxa were chosen according to the results of previous published data [8,9]. Bayesian inference (BI) and Maximum-likelihood (ML) analyses of the sequence dataset were performed at the CIPRES Science Gateway [10], using MrBayes 3.2.7a [11] and RAxML 8.2.12 [12], respectively. The best fit model of DNA evolution was obtained using jModelTest 2.1.10 [13] with the Akaike Information Criterion (AIC). The Akaike-supported model, the base frequency, the proportion of invariable sites, and the gamma distribution shape parameters and substitution rates in the AIC were then used in phylogenetic analyses. BI analysis under the general time reversible model with a proportion of invariable sites and a gamma-shaped distribution (GTR + I + G) was initiated with a random starting tree and run with the four Metropolis-coupled Markov chain Monte Carlo (MCMC) for 2 × 10^6^ generations. ML analysis was implemented under the same nucleotide substitution model as in the BI, and 1000 bootstrap replications. The topologies were used to generate a 50% majority rule consensus tree. Posterior probabilities (PP) and bootstrap support (BS) of over 70% are given on appropriate clades. The trees were visualized with the program FigTree v1.4.3 and drawn with Adobe Illustrator CC.

## 3. Results and Discussion

### 3.1. Systematics

**Figure 1:**
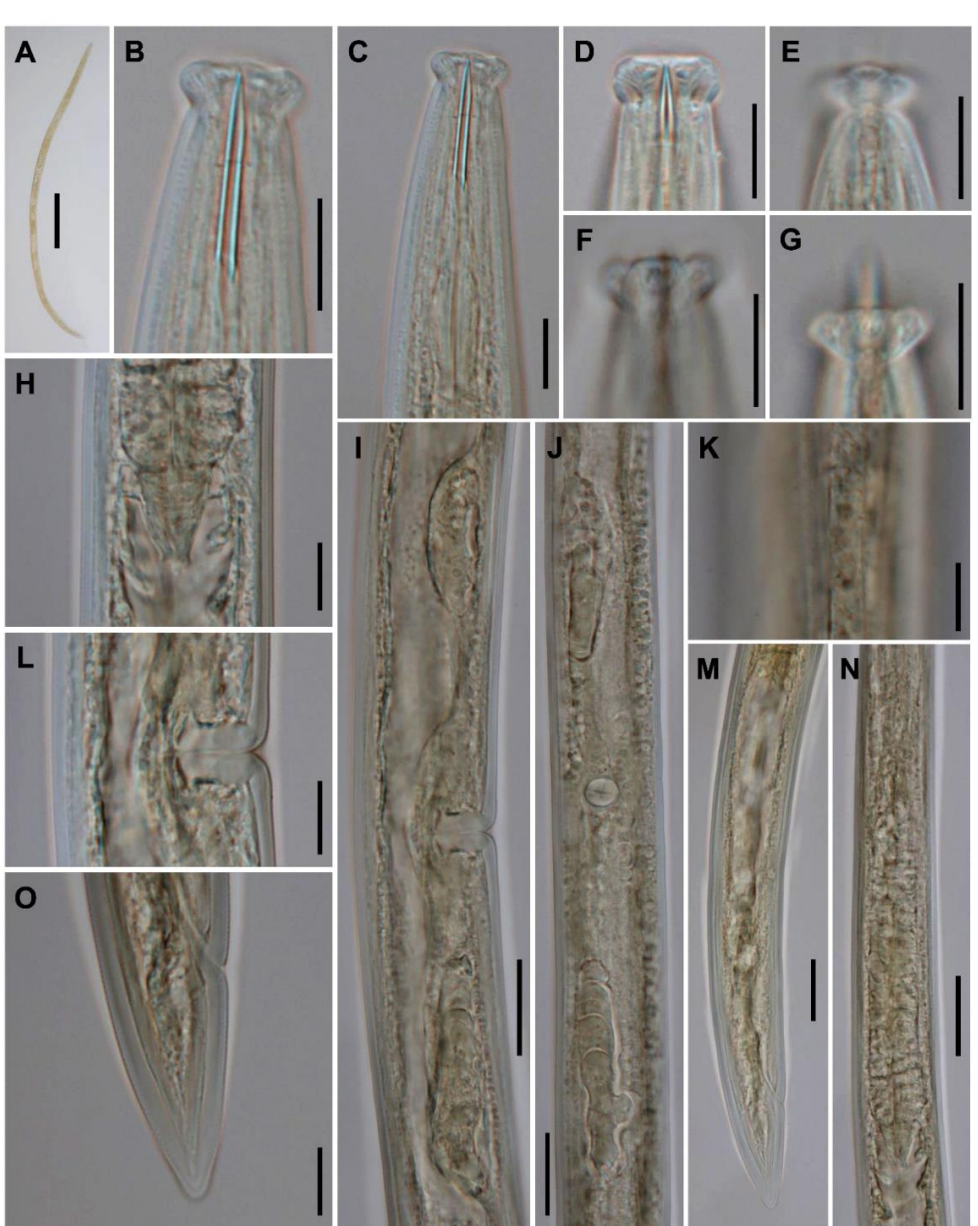
*Kochinema farodai* Baqri and Bohra, 2001 (light photomicrographs, female). (**A**) Female, entire body; (**B**,**C**) anterior region in lateral median view; (**D**) lip region in dorso-ventral median view; (**E**–**G**) lip region in surface lateral view; (**H**) pharyngo-intestinal junction; (**I**,**J**) genital system; (**K**) lateral chord; (**L**) vagina; (**M**) posterior body region; (**N**) pharyngeal expansion; (**O**) tail. Scale bars: (**A**) = 200 μm; (**B**–**H**,**K**,**L**,**O**) = 10 μm; (**I**,**J**,**M**,**N**) = 20 μm.

**Figure 2:**
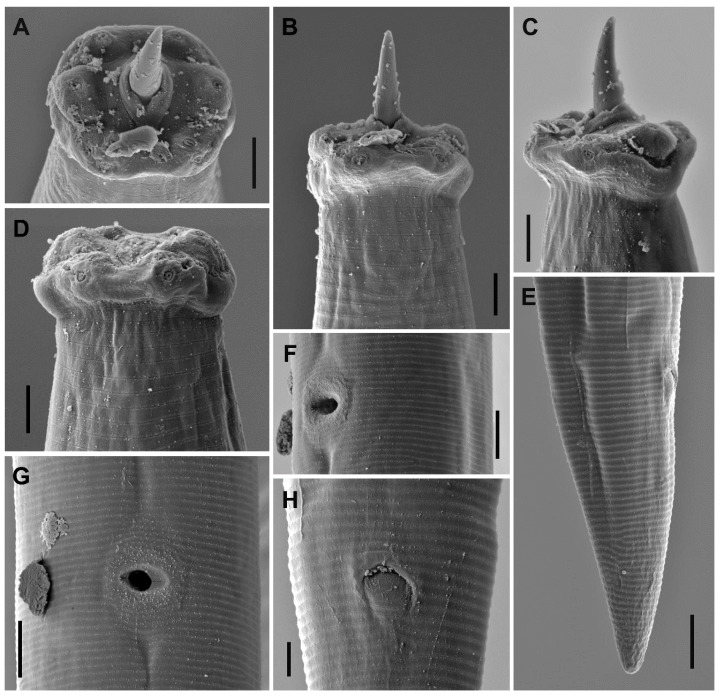
*Kochinema farodai* Baqri and Bohra, 2001 (SEM, female). (**A**) Lip region in frontal view; (**B**) lip region in ventral view; (**C**) lip region in sublateral view; (**D**) lip region in dorsal view; (**E**) caudal region in lateral view; (**F**) vulval region in lateral view; (**G**) vulval region in ventral view; (**H**) anus, ventral view. Scale bars: (**A**–**D**,**H**) = 2 μm; (**E**–**G**) = 5 μm.

*Kochinema farodai* Baqri and Bohra, 2001 (Figure 1 and Figure 2).

#### 3.1.1. Material Examined

Seven females from one location, in variable (in general acceptable) state of preservation.

#### 3.1.2. Morphometrics

See Table 1.

#### 3.1.3. Description

Female. Very slender (*a* = 41–45) nematodes of medium size, 1.13–1.30 mm long. Habitus almost straight or slightly curved ventrad upon fixation. Cuticle two-layered, 1.0 µm thick at anterior region, 1.5 µm in mid-body, and 2.5–3.0 µm on tail; outer layer thin and bearing fine transverse striation throughout the body; inner layer thicker than the outer layer. Lateral chord 5–7 µm broad or 17–24% of mid-body diameter. Body pores often obscure under LM. Lip region offset by a distinct depression and wider than adjacent body part, 2.9–3.1 times as wide as high, and 37–42% of body diameter at neck base. Dorsal and ventral lips mostly amalgamated and separated from the lateral ones, due to the amphidial aperture morphology. Lateral lips smaller than dorsal and ventral ones, due to the amphidial aperture position. Labial and cephalic papillae pore-like and non-protruding. Oral field ellipsoid and separated from adjacent part by a marked incisure, oral aperture a dorso-ventral slit, the lip region hence showing a biradial symmetry. Amphid fovea stirrup-shaped, its opening located on lateral lip and distinctly curved posteriad (as seen under SEM), and 4.5 µm broad or 41–43% of lip region diameter. Cheilostom nearly cylindrical, with no specialization. Odontostyle slender and longer (1.6–1.7 times) than lip region diameter, 12.0–12.9 times longer than wide, and 1.34–1.60% of total body length; aperture 4.5 µm or 25–26% of its total length. Guiding ring simple, located at 9–10 µm or 0.9 times of the lip region diameter from the anterior end. Odontophore rod-like, 1.1–1.2 times of the odontostyle long. Pharynx consisting of a slender but muscular anterior section enlarging very gradually in the posterior, the expansion 5.9–7.4 times as long as wide, 3.6–4.1 times of the corresponding body diameter, and occupies 38–41% of total neck length; gland nuclei obscure in the specimens examined. Nerve ring at 95–111 µm or 37–42% of total neck length from the anterior end. Pharyngo-intestinal junction surrounded by a ring-like structure. Cardia conical to rounded. Genital system didelphic–amphidelphic, with both branches equally and well developed, anterior 74–80 µm (n = 2) long or 7% (n = 1) of total body length, and posterior 74–90 µm (n = 3) long or 7–8% (n = 2) of total body length. Ovaries 34–65 µm long, often not reaching the oviduct-uterus junction, oocytes arranged first in two or more rows, then in a single row. Oviduct 42–55 µm long or 1.4–2.0 times of the body diameter, and consisting of a slender portion and a small *pars dilatata* with visible lumen, a narrowing separates oviduct and uterus. Uterus a short and simple tube-like structure 19–28 µm long or 0.6–1.0 times the corresponding body diameter. Vagina extending inwards 14–16 µm or 49–53% of body diameter, with *pars proximalis* 7–10 × 7.5–9.5 µm and convergent walls surrounded by weak musculature, *pars refringens* absent, and *pars distalis* well developed, 6–7 µm long. Vulva a post-equatorial transverse slit. Prerectum 4.2–6.8 and rectum 1.3–1.4 times the anal body diameter long. Anus, as seen under SEM, a curved anteriad transverse slit. Tail conical with rounded tip, ventrally straight or slightly convex, dorsally convex; inner core nearly reaching the tail tip. Two pairs of caudal pores at the middle of tail, one subdorsal, another lateral.

Male. Unknown.

#### 3.1.4. Molecular Characterization

**Figure 3:**
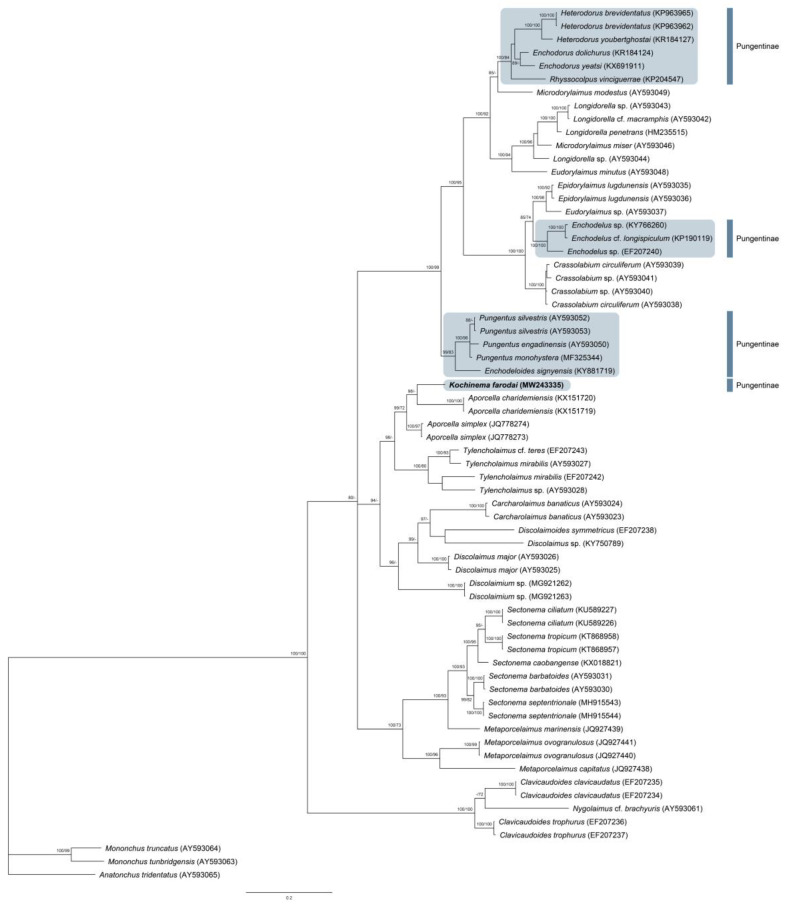
Bayesian 50% majority rule consensus tree as inferred from D2-D3 expansion segments of 28S rRNA gene sequence alignments under the GTR + I + G model. Branch support of over 70% is given for appropriate clades, and it is indicated as posterior probabilities value in Bayesian inference analysis/bootstrap value from maximum-likelihood analysis.

One sequence of the D2–D3 of 28S rRNA gene 740 bp long was obtained. The evolutionary relationships of *Kochinema faroidai* with several representatives of the order Dorylaimida are presented in Figure 3.

#### 3.1.5. Diagnosis (Based on Specimens Examined)

This species is characterized by its 1.13–1.30 mm long body, lip region offset by a distinct depression, wider than adjacent body part and 10.5–11.0 µm broad, amphidial aperture on lateral lip, odontostyle 17.5–18.0 µm at its dorsal side and 1.6–1.7 times the lip region diameter, odontostyle aperture occupying 25–26%, neck 252–298 µm long, pharyngeal expansion 98–122 µm long or 38–41% of total neck length, uterus a simple tube-like structure and 19–28 µm long or 0.6–1.1 times of the corresponding body diameter, *pars refringens vaginae* absent, vulva transverse (*V* = 51–55), female tail conical (29–37 µm, *c* = 35–44, *c′* = 1.5–1.9) with rounded terminus and inner core nearly reaching the tail tip, and male absent.

#### 3.1.6. Remarks

The Californian specimens herein studied agrees well with the original population of *K. farodai* from India, although some morphometric differences are found: longer and more slender body (*L* = 1.13–1.30 and *a* = 41–45 vs. *L* = 0.74–1.18 and *a* = 21–30, respectively), wider amphidial opening (4.5 or 41–43 vs. 3–4 μm or ca. 35–36% of lip region diameter), smaller odontostyle aperture (25–26 vs. 35% of total odontostyle length), shorter pharyngeal expansion (38–41% vs. 41–44% of total neck length), comparatively longer prerectum and rectum (4.2–6.1 and 1.3–1.4 vs. 3.2–3.5 and ca. 1.0 times anal body diameter long, respectively), and comparatively shorter tail (*c* = 35–44 vs. *c* = 26–33). Nevertheless, other more relevant taxonomical measurements and ratios are nearly identical, including lip region width (10.5–11.0 vs. 10–11 μm), odontostyle length (17.5–18.0 vs. 17–19 μm), and vulva position (*V* = 51–55 vs. *V* = 52–55). Considering that only a few specimens were studied in both populations, these differences are provisionally considered as intraspecific, reflecting geographical variability.

#### 3.1.7. Distribution

*Kochinema faroidai* was originally described from three locations from Rajasthan, India, in association to wheat (*Triticum aestivum*) and millet (*Pennisetum typhoides*). The specimens herein studied were collected from American River Parkway, River Bend Park, El Dorado County, California, USA, in association to willow (*Salix* sp.) and other trees. The finding of *K. faroidai* in California is a remarkable biogeographical novelty, as it represents the first American record of the species.

### 3.2. On the Identity and the Taxonomy of Kochinema

#### 3.2.1. Historical Outline

*Kochinema* was erected by Heyns [14] to accommodate a single nematode species, *K. proamphidum* Heyns 1963, which is characterized by the unusual position of the amphid aperture, located on the lateral lip instead of in the lip region constriction. However, when this genus was described, the author was not able to place the genus under a dorylaimid family due to its unusual pattern, and he preferred to place *Kochinema* “next to its nearest relatives in the Dorylaiminae or Tylencholaiminae”. Later, Siddiqi [15] and Argo and Van den Berg [16] added one and three new species, respectively, to the genus *Kochinema*, but they neither placed the genus under a dorylaimid family. In 1969, Siddiqi [17] classified *Kochinema* under the family Qudsianematidae Jairajpuri, 1965, but, one decade after, Darekar and Khan [18] established a new family Kochinematidae for the genera *Kochinema* and *Indokochinema* Darekar and Khan, 1979. However, Jairajpuri and Ahmad [19] considered Kochinematidae a junior synonym of the family Nordiidae Jairajpuri and Siddiqi, 1964. Additional species were added to this genus by Jana and Baqri [20] and Baqri and Bhora [21] increasing the number of known species of *Kochinema* up to eight. The taxonomy of *Kochinema* has not suffered any posterior modification or update, and only additional data of a population of *K. secutum* from the Galápagos by De Ley and Coomans [22] and a population of *K. tenue* from Iran by Vazifeh et al. [23] have been reported during the last decade.

#### 3.2.2. General Morphology

Body size and shape. The genus *Kochinema* includes small to medium size nematodes, whose body length ranges from 0.74 to 1.60 mm, and seldom exceed 1.30 mm long, with only one species having a body length larger than 1.30 mm, *K. longum* Argo and Van den Berg, 1971 (body length 1.30–1.60 mm). *Kochinema* species in general are moderately slender to very slender (*a*-ratio = 19–59), very often between 20 and 40, and with only one species having an extremely slender body, *K. longum* (*a* = 48–59).

Cuticle. Typical dorylaimoid, that consists in a two-layered cuticle. The outer layer is thin, with constant thickness throughout the body, and bearing fine transverse striation. The inner layer thicker than the outer layer, more distinct at posterior body region, lacking any special differentiation.

**Figure 4:**
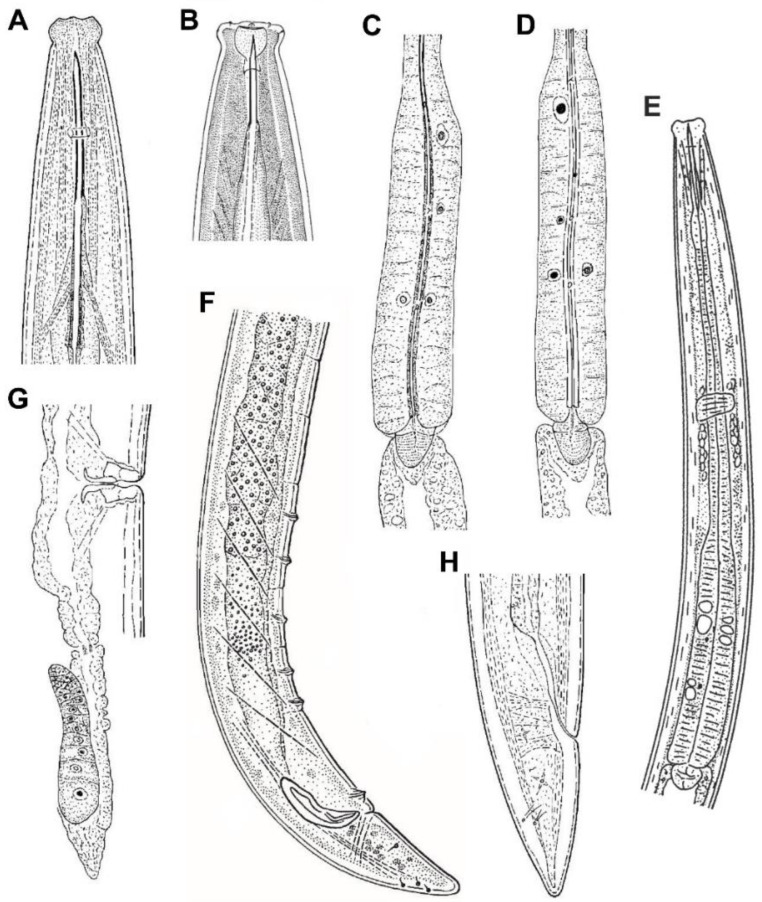
General morphology of *Kochinema* species. (**A**,**B**) Anterior region of *K. caudatum* and *K. secutum*, respectively; (**C**,**D**) pharyngeal expansion of *K. farodai* and *K. caudatum*; (**E**) neck of *K. secutum*; (**F**) male, posterior body region of *K. proamphidum*; (**G**) female, posterior genital branch of *K. caudatum*; (**H**) female tail of *K. caudatum*. (**A**,**D**,**G**), H after Baqri and Bohra, 2001. (**B**): After Siddiqi, 1965. (**C**): After Baqri and Bohra, 2001. (**E**): After De Ley and Coomans, 2013. (**F**): After Heyns, 1963.

Lip region. It is offset by a distinct depression and wider than the adjacent body part (Figure 1B and Figure 4A,B). Anterior margin somewhat rounded (Figure 4A). Lateral lips smaller and separated from dorsal and ventral ones which are mostly amalgamated, and their labial and cephalic papillae are pore-like and non-protruding. Oral aperture a dorso-ventral slit, oral field ellipsoid, and separated from adjacent part by a marked incisure (Figure 2A), hence resulting in a biradial symmetry of the lip region.

Amphids. The unusual location of the amphid aperture is the main morphological feature of *Kochinema*. The anterior position of amphids, whose opening is located on the lateral lip (Figure 2C), is very unusual in dorylaims. However, this remarkable feature also occurs in the genus *Ecumenicus* Thorne, 1974. Among dorylaimid species, the position of amphids is usually postlabial—i.e., situated behind the lateral lips—with their aperture situated at the junction of the lip region with the adjacent body part.

Odontostyle. Slender, straight (Figure 1B and Figure 4B) or slightly arcuate dorsally (Figure 4A), and with a small aperture. It is possible to separate it into two species-groups based in the odontostyle length: (i) *Kochinema* species characterized by longer odontostyle, which is 31–41 µm long, includes three species, namely *K. caudatum* Baqri and Bohra, 2001; *K. crassatum* Argo and Van den Berg, 1971; and *K. proamphidum* Heyns, 1963 and (ii) *Kochinema* species characterized by shorter odontostyle, which is 13-22 µm long, includes four species, namely *K. farodai*; *K. longum*; *K. secutum* Siddiqi, 1965; and *K. tenue* Argo and Van den Berg, 1971. The aperture occupies 10–35% of the total odontostyle length.

Guiding ring. The currently known *Kochinema* species included species characterized by having simple, weakly refractive, guiding ring (*K. farodai* and *K. secutum*) and species characterized by having double guiding ring (*K. caudatum*, *K. crassatum*, *K. longum*, *K. proamphidum*, and *K. tenue*; see note on the latter species below).

Pharynx. It follows the typical dorylaimid pattern, with gradual enlargement and comparatively short pharyngeal expansion, which occupies around two-fifths (36–44%) of the total neck length (Figure 4E). The rather anterior location of S_2_N gland nuclei (S_2_N = 83–88) in the pharyngeal expansion is remarkable (Figure 4C–E).

Female genital system. Invariably didelphic–amphidelphic (Figure 1I,J). Precise details of the genital system remain unknown for some species. The system is always well developed, each genital branch consisting of an ovary, an oviduct, and a uterus. The latter is nearly always a short tube-like structure (usually less than two times the corresponding body diameter) without other special differentiation (Figure 4G). The *pars refringens vaginae* is completely absent, and the *pars distalis vaginae* is well developed (Figure 1L and Figure 4G).

Male genital system and secondary structures. Four out of the seven valid species of the genus are male known. As usual, the male genital system, always diorchic, does not show any relevant variation affecting its gonads, ducts, and secondary sexual structures. The spicules range from 29 to 46 µm long, the ventromedian supplements (5–7) are spaced, and a wide hiatus is always present (Figure 4F).

Caudal region. It is similar in both sexes, conical with rounded terminus, and usually longer than anal/cloacal body diameter (Figure 1O and Figure 4F,H), *c′* = 1.4–2.0, except in *K. crassatum* (*c′* = 1.0 in females). Its inner core ending near the tail tip; hence, a hyaline portion is not distinguishable (Figure 1O and Figure 4H).

### 3.3. On the Phylogeny of Kochinema

This contribution provides the first molecular study of the genus *Kochinema*, and therefore, the intrageneric variation of the genus cannot be analyzed at this moment, a matter very interesting to study using molecular data, especially due to some remarkable morphological differences found among *Kochinema* species—for instance, the guiding ring nature (see above). However, the new molecular data herein obtained allow us to study the intergeneric relationships of *Kochinema* as well as to show its phylogenetic position among dorylaimid taxa. The derived phylogenetic tree (Figure 3), inferred from D2–D3 expansion segments of 28S rRNA gene, shows that *Kochinema farodai* is clustered together with members of the families Aporcelaimidae (genus *Aporcella* Andrássy, 2002) and Tylencholaimidae (genus *Tylencholaimus* De Man, 1876) in a well-supported clade (posterior probabilities BI: 98 and ML: 72). In addition, this clade is clustered inside of a larger and well-supported clade (posterior probabilities BI: 94), which is formed by a heterogeneous group of taxa, from a morphological point of view. This larger clade includes representatives of the family Qudsianematidae (genera *Discolaimium* Thorne, 1939; *Discolaimus* Cobb, 1913; *Discolaimoides* Heyns, 1963; and *Carcharolaimus* Thorne, 1939), in which all of them share one remarkable morphological pattern—the absence of *pars refringens vaginae*. Moreover, the lip region morphology of *Kochinema*, wider than adjacent body part, also resembles the lip region pattern found in some of the members of Discolaiminae Siddiqi, 1969—a subfamily of Qudsianematidae. The phylogenetic tree obtained also shows that the genus *Kochinema* does not share a recent common ancestor with other member of the family Nordiidae. Furthermore, these results could also confirm previous results which suggested that the D2–D3 gene might not be useful in elucidating the internal evolutionary relationships among members of the suborder Dorylaimina [4].

On the other hand, the results obtained also confirm that subfamily Pungentinae Siddiqi, 1964, and the family Nordiidae Jairajpuri and Siddiqi, 1964, are not natural (monophyletic) taxa (see Figure 3), and agree with previously reported phylogenetic studies of this family [24,25]. The polyphyly of several families (for instance, Qudsianematidae, Dorylaimidae, Nordiidae, and Aporcelaimidae) of the superfamily Dorylaimoidea were previously reported [4,8,26], a matter that should be studied using an integrative approach and analyzing several DNA genes—for instance, ITS and Cox1—to update the current taxonomy of Dorylaimoidea, which is far from satisfactory.

### 3.4. Updated Taxonomy of Kochinema

#### 3.4.1. Diagnosis

Small- to medium-sized nematodes 0.74–1.60 mm long. Cuticle two-layered. Lip region offset by a distinct depression and wider than adjacent body part; oral aperture a dorsoventral slit, and oral field ellipsoid. Amphid in anterior position, with its aperture situated on lateral lip. Odontostyle slender, straight or slightly arcuate dorsally, longer than lip region diameter, and with a small aperture. Guiding ring simple or double. Pharynx distinctly muscular, enlarging very gradually, with the expanded posterior section occupying ca. two-fifths of the total neck length. Female genital system didelphic-amphidelphic; uterus a short tube-like structure; *pars refringens vaginae* absent; vulva a post-equatorial transverse slit, *V* = 51–59. Tail similar in both sexes, conical with rounded terminus, equal or longer than anal/cloacal body diameter, and with its inner core ending near the tail tip. Spicules dorylaimoid, slender. Ventromedian supplements five to seven, spaced and showing hiatus.

#### 3.4.2. List of Species

Type species:

Kochinema proamphidum Heyns, 1963.

Other species:

*K. caudatum* Baqri and Bohra, 2001;

*K. crassatum* Argo and Van den Berg, 1971;

*K. farodai* Baqri and Bohra, 2001;

*K. longum* Argo and Van den Berg, 1971;

*K. secutum* Siddiqi, 1965;

*K. tenue* Argo and Van den Berg, 1971;

Species incertae sedis;

*K. longicaudatum* Jana and Baqri, 1985.

#### 3.4.3. Key to Species Identification

1- Longer odontostyle, more than 30 μm2
- Shorter odontostyle, equal or less than 22 μm42- Longer odontostyle, 41 μm long; and comparatively shorter female tail, *c′* = 1.0crassatum
- Shorter odontostyle, up to 38 μm; and comparatively longer female tail, *c′* ≥ 1.633- Odontostyle 35–38 μm long or 2.6–2.7 times the lip region diameter; comparatively longer neck in females, *b* = 3.1–4.0; comparatively shorter female tail, *c′* = 1.6–1.7; and male absentcaudatum
- Odontostyle 31–35 μm long or 2.0 times the lip region diameter; comparatively shorter neck in females, *b* = 4.0–4.9; comparatively longer female tail, *c′* = 2.0; and male presentproamphidum4- Body 1.36–1.60 mm long and very slender, *a* = 48–59; vulva more posterior, *V* = 59longum
- Body up to 1.30 mm long and less slender, *a* up to 45; vulva less posterior, *V* = 51–5655- Shorter odontostyle, 13–15 μm long; and male presentsecutum
- Longer odontostyle, equal or more than 15 μm long; and male absent66- Lip region 9–10 μm broad; odontostyle aperture occupying 20% of total odontostyle length; and double guiding ringtenue
- Lip region 10–11 μm broad; and odontostyle aperture occupying 25–35% of total odontostyle length; and simple guiding ringfarodai

#### 3.4.4. Notes about Some Species

*K. longicaudatum*: some of the main morphological features of this species does not fit the *Kochinema* pattern: vulva pre-equatorial (*V* = 42–44), presence of *pars refringens vaginae*, and long filiform female tail (*c′* = 12–14). Furthermore, there are contradictory or erroneous data for the odontostyle length, which according the original description of the species, is longer (1.2–1.3 times) than lip region diameter; nevertheless, Figure 2B,D show that odontostyle is slightly shorter than lip region diameter. Additional information is needed to clarify the true identity of this species—the reason why it is considered herein as a *species incertae sedis*.

*K. farodai*: considering the new data herein obtained for this species, *K. farodai* is morphologically and morphometrically very similar to *K. secutum* and *K. tenue*. This very similar group of species can only be distinguished based on minor morphometrics differences, and they might be conspecific. However, without examining the type material of these species, a separate status is provisionally maintained for them.

*K. secutum*: this species was originally described by Siddiqi [15] based on only one female specimen from India. More recently, De Ley and Coomans [22] identified a population from the Galapagos as *K. secutum*, including the description of a single male specimen. These authors also noted that *K. secutum* is very similar to *K. tenue*. Currently, both species only can be distinguished in odontostyle length (13–15 μm in *K. secutum* vs. 15–18 μm in *K. tenue*) and the guiding ring nature (simple vs. double).

*K. tenue*: the original description of the species stated that the guiding ring is double; however, this detail cannot be confirmed in the original illustration of the species, since in the specimen illustrated in Figure 1A the odontostyle protrudes. If this feature is confirmed for this species, the guiding ring nature is the main morphological feature to distinguish *K. tenue* from its closest species (*K. secutum* and *K. farodai*).

Table S1 (Supplementary Material) provides a compendium of *Kochinema* species with taxonomically important morphometrics characters and information on its current distribution.

## Figures and Tables

**Table 1:** Morphometrics of *Kochinema farodai* Baqri and Bohra, 2001, from California. Measurements in μm (except L, in mm), and in the form: mean ± standard deviation (range).

Population		El Dorado County, California, USA
Character *	n	7 Females
L		1.23 ± 0.07 (1.13–1.30)
a		42.7 ± 1.8 (41–45)
b		4.7 ± 0.4 (4.3–5.1)
c		38.6 ± 4.1 (35–44)
c′		1.8 ± 0.2 (1.5–1.9)
V		52.9 ± 1.5 (51–55)
Lip region diameter		10.9 ± 0.2 (10.5–11.0)
Odontostyle length		17.9 ± 0.2 (17.5–18.0)
Odontophore length		19.8 ± 0.8 (19–21)
Guiding ring from anterior end		9.5 ± 0.4 (9–10)
Neck length		266 ± 21.3 (252–298)
Pharyngeal expansion length		105 ± 9.6 (98–122)
Diameter at neck base		27.6 ± 1.2 (26.0–29.5)
at midbody		28.9 ± 1.1 (27.5–30.0)
at anus		18.1 ± 0.9 (17–19)
Prerectum length		102 ± 21.5 (80–123)
Rectum length		24.5 ± 1.5 (22–26)
Tail length		32.4 ± 3.1 (29–37)

* Abbreviations: a = body length/greatest body diameter; b = body length/distance from anterior end to pharyngo-intestinal junction; c = body length/tail length; c′ = tail length/tail diameter at anus or cloaca; L = overall body length; n = number of specimens on which measurements are based; V = distance from body anterior end to vulva expressed as percentage (%) of the body length.

## Supplementary Materials

The following are available online at https://www.mdpi.com/2076-2615/10/12/2300/s1, Table S1: Main morphometrics and distribution data of valid species belonging to *Kochinema* Heyns, 1963. All measurements are in μm, except L, in mm.

## References

[1] Andrássy I. (2009). Free-Living Nematodes of HUNGARY, III (Nematoda errantia). Pedozoologica Hungarica No. 5.

[2] Barker K.R., Barker K.R., Carter C.C., Sasser J.N. (1985). Nematode extraction and bioassays. An Advanced Treatise on Meloidogyne, Volume II. Methodology.

[3] Siddiqi M.R. (1964). Studies on *Discolaimus* spp. (Nematoda: Dorylaimidae) from India. Z. Zool. Syst. Evol..

[4] Álvarez-Ortega S., Peña-Santiago R. (2016). *Aporcella charidemiensis* sp. n. (Dorylaimida: Aporcelaimidae) from the southern Iberian Peninsula, with comments on the phylogeny of the genus. Nematology.

[5] Álvarez-Ortega S., Subbotin S.A., Peña-Santiago R. (2013). Morphological and molecular characterisation of *Aporcelaimellus simplex* (Thorne & Swanger, 1936) Loof & Coomans, 1970 and a new concept for *Aporcella* Andrássy, 2002 (Dorylaimida: Aporcelaimidae). Nematology.

[6] Subbotin S.A., Sturhan D., Chizhov V.N., Vovlas N., Baldwin J.G. (2006). Phylogenetic analysis of Tylenchida Thorne, 1949 as inferred from D2 and D3 expansion fragments of the 28S rRNA gene sequences. Nematology.

[7] Thompson J.D., Gibson T.J., Plewniak F., Jeanmougin F., Higgins D.G. (1997). The ClustalX windows interface: Flexible strategies for multiple sequence alignment aided by quality analysis tools. Nucleic Acids Res..

[8] Holterman M., Rybarczyk K., van den Elsen S., van Megen H., Mooyman P., Peña-Santiago R., Bongers T., Bakker J., Helder J. (2008). A ribosomal DNA-based framework for the detection and quantification of stress-sensitive nematode families in terrestrial habitats. Mol. Ecol. Resour..

[9] Álvarez-Ortega S., Peña-Santiago R. (2019). Morphology, phylogeny and taxonomy of the genus *Sectonema* (Nematoda, Aporcelaimidae). Zool. Scr..

[10] Miller M., Pfeiffer W., Schwartz T. Creating the CIPRES Science Gateway for inference of large phylogenetic trees. Proceedings of the Gateway Computing Environments Workshop (GCE).

[11] Ronquist F., Teslenko M., van der Mark P., Ayres D.L., Darling A., Höhna S., Larget B., Liu L., Suchard M.A., Huelsenbeck J.P. (2012). MrBayes 3.2: Efficient Bayesian phylogenetic inference and model choice across a large model space. Syst. Biol..

[12] Stamatakis A. (2014). RAxML version 8: A tool for phylogenetic analysis and post-analysis of large phylogenies. Bioinformatics.

[13] Darriba D., Taboada G.L., Doallo R., Posada D. (2012). jModelTest 2: More models, new heuristics and parallel computing. Nat. Methods.

[14] Heyns J. (1963). New species of the superfamily Dorylaimoidea (Nemata) from South Africa soil with a description of the genus *Kochinema*. S. Afr. J. Agric. Sci..

[15] Siddiqi M.R. (1965). Seven new species of Dorylaimoidea (Nematoda) from India, with description of *Lenonchium* n. gen. and *Galophinema* n. gen. Proc. Helm. Soc. Wash..

[16] Argo A.O., Van den Berg E. (1971). Three new species of the genus *Kochinema* (Nematoda: Dorylaimoidea) from South Africa. Phytophylactica.

[17] Siddiqi M.R. (1969). *Crateronema* n. gen. (Crateronematidae n. fam.), *Poronemella* n. gen. (Lordellonematinae n. sub. fam) and *Chrysonemoides* n. gen. (Chrysonematidae n. fam.) with a revised classification of Dorylaimoidea (Nematoda). Nematologica.

[18] Darekar K.S., Khan E. (1979). Soil and plant-parasitic nematodes from Maharashtra, India. VII. *Indokochinema conicauda* n. gen., n. sp. and Kochinematidae n. fam. (Dorylaimida: Nematoda). Indian J. Nematol..

[19] Jairajpuri M.S., Ahmad W. (1992). Dorylaimida—Free-Living, Predaceous and Plant-Parasitic Nematodes.

[20] Jana A., Baqri Q.H. (1985). Nematodes from West Bengal (India) XV. On the species of some rare genera having narrow odontostyle of the super family Dorylaimoidea (Dorylaimida). Bull. Zool. Surv. India.

[21] Baqri Q.H., Bohra P. (2001). Nematodes from Rajasthan, India. 1. Six new species of Dorylaimida. Nematology.

[22] De Ley P., Coomans A. (2013). Terrestrial nematodes from the Galápagos Archipelago. 11. The morphology of three rare Dorylaimoidea (Dorylaimida). J. Nematode Morphol. Syst..

[23] Vazifeh N., Niknam G., Jabbari H. (2018). Report of five dorylaimid nematodes from East Azerbaijan province, Iran. Iran. J. Plant Pathol..

[24] Pedram M., Pourjam E., Robbins R.T., Ye W., Peña-Santiago R. (2011). Description of *Rhyssocolpus vinciguerrae* sp. n. (Dorylaimida: Nordiidae) from Iran and the first molecular study of this genus. Nematology.

[25] Peña-Santiago R., Guerrero P., Liébanas G., García M.C., Palomeque T., Lorite P. (2015). Characterisation of an Iberian population of *Rhyssocolpus iuventutis* Andrássy, 1971 (Dorylaimida: Nordiidae), with a revised taxonomy of the genus. Nematology.

[26] Elshishka M., Lazarova S., Radoslavov G., Hristov P., Peneva V.K. (2015). New data on two remarkable Antarctic species *Amblydorylaimus isokaryon* (Loof, 1975) Andrássy, 1998 and *Pararhyssocolpus paradoxus* (Loof, 1975), gen. n., comb. n. (Nematoda: Dorylaimida). ZooKeys.

