# Supplementary material for: Morphological and Molecular Characterization of Kochinema farodai Baqri and Bohra, 2001 (Dorylaimida: Nordiidae) from California, with the First Molecular Study and an Updated Taxonomy of the Genus
Source: Animals (Basel). 2020 Dec 4;10(12):2300. doi: 10.3390/ani10122300 (PMC7761927; doi:10.3390/ani10122300)
Supplement: Supplementary file 1 [file animals-10-02300-s001.pdf]

animals

MDPI

Article

Morphological and Molecular Characterization of *Kochinema farodai* Baqri and Bohra, 2001 (Dorylaimida: Nordiidae) from California, with the First Molecular Study and an Updated Taxonomy of the Genus

Sergio Álvarez-Ortega

Departamento de Biología y Geología, Física y Química Inorgánica, Universidad Rey Juan Carlos, Campus de Móstoles, 28933 Madrid, Spain; sergio.aortega@urjc.es

Received: 16 November 2020; Accepted: 30 November 2020; Published: date

Table S1. Main morphometrics and distribution data of valid species belonging to *Kochinema* Heyns, 1963. All measurements are in μm, except L, in mm.

	Character																
	Species	n	L	a	b	c	c'	V	Lrd	Odont.	Neck	Ph. exp.	Tail	Spicul.	Ve. sup.	Geog. Dis.	Ref.
**1**	*caudatum*	6♀♀	0.81–0.95	25–30	3.1–4.0	24–27	1.6–1.7	54–57	13–14	35–38	261 *	37–40%	32–35	-	-	India	[15]
**2**	*crassatum*	4♀♀	0.93–1.28	19–24	4.3–5.0	22–24	1.0	55–58	17 *	41	251 *	-	45 *	-	-	South Africa	[17]
		♂	1.30	21	4.7	23	1.5	-	-	-	277 *	-	57 *	46	6–7		
**3**	*farodai*	7♀♀	0.74–1.18	21–30	4.1–4.5	26–33	1.4–1.6	52–55	10–11	17–19	191–234	41–44%	28–35	-	-	India	[15]
		7♀♀	1.13–1.30	41–45	4.3–5.1	35–44	1.5–1.9	51–55	10.5–11	17.5–18	252–298	38–41%	29–37	-	-	California, USA	Present Paper
**4**	*longum*	2♀♀	1.36–1.60	48–59	5.6	41–44	1.5	59	11.5 *	18–22	243–286	-	33–37*	-	-	South Africa	[17]
		♂	1.30	52	7.6	36	2.0	-	-	-	171 *	-	36 *	44	7		
**5**	*proamphidum*	3♀♀	0.82–0.98	23–29	4.2–4.6	20–24	2.0	56–59	-	-	-	*ca* 40% *	-	-	-	South Africa	[1]
		2♂♂	0.86–1.22	26–27	4.2–4.5	21–24	-	-	-	-	-	-	-	-	5–7		
		5♀♀	0.89–1.22	22–28	4.0–4.9	20–24	-	54–58	-	31–35	-	-	-	-	-	South Africa	[17]

		9♂♂	0.90–1.17	24–32	3.9–5.6	20–27	-	-	-	31–34	-	-	-	35–39	-		
**6**	*secutum*	♀	1.03	38	4.7	28	2.0	53	11	15	219 *	40%	37 *	-	-	India	[16]
		10♀♀	0.74–0.98	33–39	4.0–4.4	27–32	1.6–2.0	51–56	8.5–10	13–15	188–231	*ca* 40%	27–34	-	-	Galápagos	[22]
		♂	0.91	38	4.3	29	1.6	-	-	14	214	36% *	32	29	5		
**7**	*tenue*	5♀♀	0.81–0.87	27–34	4.2–4.4	24–27	1.5	55	10 *	17	198 *	40% *	37 *	-	-	South Africa	[17]
		9♀♀	0.80–1.11	29–39	3.8–4.7	25–35	1.5–1.8	51–56	9–10	15–18	193–234	-	26–32	-	-	Iran	[23]

Abbreviations for columns: Lrd: lip region diameter. Odont.: odontostyle length. Ph. exp.: pharyngeal expansion length. Spicul.: spicule length. Ve. sup.: number of ventromedian supplements. Geog. dis.: geographical distribution. - Information not available. * Calculated from original description.

Publisher's Note: MDPI stays neutral with regard to jurisdictional claims in published maps and institutional affiliations

© 2020 by the authors. Submitted for possible open access publication under the terms and conditions of the Creative Commons Attribution (CC BY) license (http://creativecommons.org/licenses/by/4.0/).
